# ENDOCRINOLOGY IN THE TIME OF COVID-19: Management of adrenal insufficiencyThis manuscript is part of a commissioned series of urgent clinical guidance documents on the management of endocrine conditions in the time of COVID-19. This clinical guidance document underwent expedited open peer review by Stefanie Hahner (Universitätsklinikum Würzburg, Würzburg, Germany), Ad R M M Hermus (University Medical Centre, Nijmegen, The Netherlands), Andrea Isidori (Sapienza University of Rome, Rome, Italy), and Jeremy W Tomlinson (Oxford Centre for Diabetes, Endocrinology and Metabolism, Churchill Hospital, Oxford, UK)

**DOI:** 10.1530/EJE-20-0361

**Published:** 2020-07-01

**Authors:** Wiebke Arlt, Stephanie E Baldeweg, Simon H S Pearce, Helen L Simpson

**Affiliations:** 1 Institute of Metabolism and Systems Research, College of Medical and Dental Sciences, University of Birmingham, Birmingham, UK; 2 Department of Endocrinology, Queen Elizabeth Hospital Birmingham, University Hospitals Birmingham NHS Foundation Trust, Birmingham, UK; 3 Department of Diabetes and Endocrinology, University College London Hospitals NHS Foundation Trust, London, UK; 4 Department of Medicine, University College London, London, UK; 5 Translational and Clinical Research Institute, Newcastle University, Newcastle upon Tyne, UK; 6 Department of Endocrinology, Newcastle upon Tyne Hospitals NHS Foundation Trust, Newcastle upon Tyne, UK; 7 Genetics and Genomic Medicine, University College London Great Ormond Street Institute for Child Health, London, UK

## Abstract

We provide guidance on prevention of adrenal crisis during the global COVID-19 crisis, a time with frequently restricted access to the usual level of healthcare. Patients with adrenal insufficiency are at an increased risk of infection, which may be complicated by developing an adrenal crisis; however, there is currently no evidence that adrenal insufficiency patients are more likely to develop a severe course of disease. We highlight the need for education (sick day rules, stringent social distancing rules), equipment (sufficient glucocorticoid supplies, steroid emergency self-injection kit) and empowerment (steroid emergency card, COVID-19 guidelines) to prevent adrenal crises. In patients with adrenal insufficiency developing an acute COVID-19 infection, which frequently presents with continuous high fever, we suggest oral stress dose cover with 20 mg hydrocortisone every 6 h. We also comment on suggested dosing for patients who usually take modified release hydrocortisone or prednisolone. In patients with adrenal insufficiency showing clinical deterioration during an acute COVID-19 infection, we advise immediate (self-)injection of 100 mg hydrocortisone intramuscularly, followed by continuous i.v. infusion of 200 mg hydrocortisone per 24 h, or until this can be established, and administration of 50 mg hydrocortisone every 6 h. We also advise on doses for infants and children.

## Introductory remarks

This guidance has been drawn up to inform clinicians and healthcare staff in their quest to provide guidance on the optimal management of patients with adrenal insufficiency under the circumstances of an acute global healthcare capacity crisis due to COVID-19, the viral illness caused by the novel corona virus SARS-CoV-2.

For the purposes of this guidance, we define primary adrenal insufficiency (PAI) as all patients with loss of function of the adrenal itself, mostly either due to autoimmune adrenalitis, that is, Addison's disease described by the eponymous Thomas Addison, or other causes including congenital adrenal hyperplasia, bilateral adrenalectomy and adrenoleukodystrophy. The overwhelming majority of PAI patients suffer from both glucocorticoid and mineralocorticoid deficiency. Our guidance similarly applies to patients with secondary adrenal insufficiency (SAI) due to hypothalamic or pituitary disease; these patients typically suffer from glucocorticoid deficiency, in the majority in combination with deficiency of other hypothalamic-pituitary axes. Similarly, the same precautionary rules apply to patients with tertiary adrenal insufficiency due to chronic exogenous glucocorticoid therapy for treatment of other conditions. Patients at risk of tertiary adrenal insufficiency are those treated with prednisolone-equivalent doses of greater than 5 mg daily for longer than 4 weeks.

## Are patients with adrenal insufficiency at an increased risk of COVID-19?

Yes, patients with adrenal insufficiency are at increased risk of COVID-19; they are at an increased risk of catching this infection and they have a higher risk of complications due to the potential for an adrenal crisis to be triggered by the infection. There is currently no evidence, however, suggestive of a higher likelihood of a severe course of disease in patients with AI falling ill with COVID-19.

- *Risk of adrenal crisis during acute illness:* Patients with adrenal insufficiency are at risk to develop a potentially life-threatening adrenal crisis if experiencing major stress, such as an acute illness. This requires administration of increased doses of glucocorticoid replacement to prevent and, if already in progress, treat the adrenal crisis (1, 2, 3). Adrenal crises are regularly observed in patients with PAI and SAI (4, 5, 6, 7) and contribute to the observed increased mortality in these patients.
- *Increased risk of infections in adrenal insufficiency:* Patients with PAI including Addison's disease and congenital adrenal hyperplasia have been shown to be at an increased risk of infections (8, 9, 10); this has also been shown for patients with SAI due to hypothalamic-pituitary disease (11). Furthermore, respiratory infections have been shown to contribute to the increased mortality observed in patients with PAI (12, 13). In addition, patients with PAI have been shown to have significantly decreased natural killer cell cytotoxicity (14), an important function of the innate immune system in fighting viral infections. Therefore, patients with PAI can be assumed to be at an increased risk of infection with COVID-19. Patients receiving supraphysiologic, immunosuppressive doses of exogenous glucocorticoids for the treatment of another condition are at even higher risk of infection.

## How should we manage patients with an established diagnosis of adrenal insufficiency?

### A. Prevention mode

All patients with established adrenal insufficiency should be provided with adequate self-management support to enable them to manage their conditions adequately and safely. Self-management support can be facilitated and communicated by mailshot, video, text, email phone call or videoconferencing, as appropriate. This should follow the 3E framework for self-management support (Educate, Equip and Empower).

#### Educate

- Ensure that all patients (and their families/partners/carers) are educated in the use of ‘the sick day rules’, that is, the need to increase their usual glucocorticoid replacement dose during intercurrent illness and the need to self-inject hydrocortisone and call for emergency medical assistance when the oral medication cannot be reliably absorbed due to vomiting or diarrhoea and/or the presence of severe and major illness or trauma. General sick day rules for patients with adrenal insufficiency are described in detail in recently published clinical guidelines (15, 16, 17); see also https://endo-ern.eu/wp-content/uploads/2019/03/20190312-Stressinstructie-addisoncrisis-hydrocortison-ENG-Endo-ERN-approved.pdf. However, for the purposes of this guidance, we have revised the generic sick day rules, having in mind patients with an acute COVID-19 infection, which frequently presents with high fever over sustained periods of time (see Table 1 and section B).
- Patients with adrenal insufficiency are at increased risk of COVID-19, albeit not as high as in patients undergoing cancer treatment or taking high doses of potent immunosuppressive drugs. All patients with adrenal insufficiency should ‘observe stringent social distancing’. If they are working, they should either work from home or work under conditions that allow very stringent social distancing at all times. This means that adrenal insufficiency patients should not work in situations that do now allow them to keep their safe distance, as is the case, for example, for healthcare workers, carers and supermarket cashier staff. It will be important to provide patients with letters stating this fact to ensure their employers are informed and can adjust working conditions as appropriate.

**Table 1:** Suggested management and hydrocortisone stress dose cover in patients with adrenal insufficiency and suspected or confirmed COVID-19 infection.

Clinical scenario	Suggested management
At home	
Onset of ‘signs and symptoms suggestive of COVID-19’ (fever >38°C (>100 F), a new or continuous dry cough, sore throat, loss of sense of smell or taste, aches and pains, fatigue)	Adults and adolescents should take 20 mg hydrocortisone orally every 6 h (in children, their usual daily dose should be trebled (i.e. 3-fold increase) and administered in four equal doses every 6 h)
	Patients on modified release hydrocortisone should switch to immediate release hydrocortisone and take 20 mg orally every 6 h
	Patients on 5–15 mg prednisolone daily should take 10 mg prednisolone every 12 h; patients on oral prednisolone >15 mg should continue their usual dose but take it split into two equal doses of at least 10 mg each
	If on fludrocortisone, continue at usual dose
	Take paracetamol 1000 mg every 6 h for fever (adjust dose appropriately for infants and children)
	Rest, drink regularly and monitor how concentrated (dark) urine looks is to guide further fluid intake
	Request medical advice on the suspected COVID-19 infection
Onset of signs and symptoms of ‘clinical deterioration’ (dizziness; intense thirst; shaking uncontrollably; drowsiness, confusion, lethargy; vomiting; severe diarrhoea; increasing shortness of breath, respiratory rate >24/min, difficulty speaking	Immediately inject (patient or carer) 100 mg hydrocortisone per i.m. injection in adults and adolescents (25 mg in infants, 50 mg in school children)
	Call for emergency medical attention for treatment and transfer to hospital, consider making their own way to hospital
	If patients cannot be taken or kept in hospital, then they should take 50 mg hydrocortisone every 6 h orally at home; if possible, they should receive i.v. hydrocortisone and an isotonic saline infusion in the admissions unit
At hospital	
On regular ward or intensive care ward, irrespective of whether breathing unaided or supported by continuous positive airway pressure (CPAP) respiration or mechanically ventilated	Hydrocortisone 100 mg per iv injection in adults and adolescents, followed by continuous iv infusion of 200 mg hydrocortisone/24 h (alternatively 50 mg every 6 h per i.v. or i.m. bolus injection)
	Infants and children should receive an initial parenteral injection of 50 mg hydrocortisone/m^2^ (usually 25 mg in infants and 50 mg in children) followed by 50 mg/24 h in infants and 100 mg/24 h in children
	Pause fludrocortisone in adults
	Continuous i.v. fluid resuscitation with isotonic saline; regularly check urea and electrolytes
Recovery; improving respiratory function, reducing or normal temperature	Gradual tapering of stress dose hydrocortisone down to double regular replacement dose at time of discharge (endocrinologist to advise)
	Re-start usual fludrocortisone dose in adults when total daily hydrocortisone dose <50 mg

#### Equip

- Ensure that the patient has ‘sufficient supplies of oral glucocorticoid preparations’ (usually hydrocortisone, but also cortisone acetate, prednisolone or prednisone). Ensure that patients who usually take modified release hydrocortisone preparations have a sufficient supply of immediate release, regular oral hydrocortisone for emergency use, for example, by prescribing an extra 4-week supply of hydrocortisone 10 mg three times daily. In patients with PAI including congenital adrenal hyperplasia also ensure sufficient mineralocorticoid supplies (fludrocortisone). Consider issuing prescriptions of 3-month hydrocortisone supplies every 2 months and arrange for them to be dispensed by mail; this will ensure that the patient has access to sufficient extra glucocorticoid doses in case of intercurrent illness. Patients should understand that they must continue taking their glucocorticoid replacement under all circumstances and that there is no need to increase the dose unless they fall ill.
- Ensure that the patient is in possession of an ‘up-to-date hydrocortisone emergency self-injection kit’ and that the patient and a relative/partner/friend is confident in self-administration of the injection. Consider refreshing knowledge by talking through the procedure over the phone and providing links to training videos (https://www.addisonsdisease.org.uk/the-emergency-injection-for-the-treatment-of-adrenal-crisis and https://www.adrenals.eu/animations/how).

#### Empower

**Figure 1:**
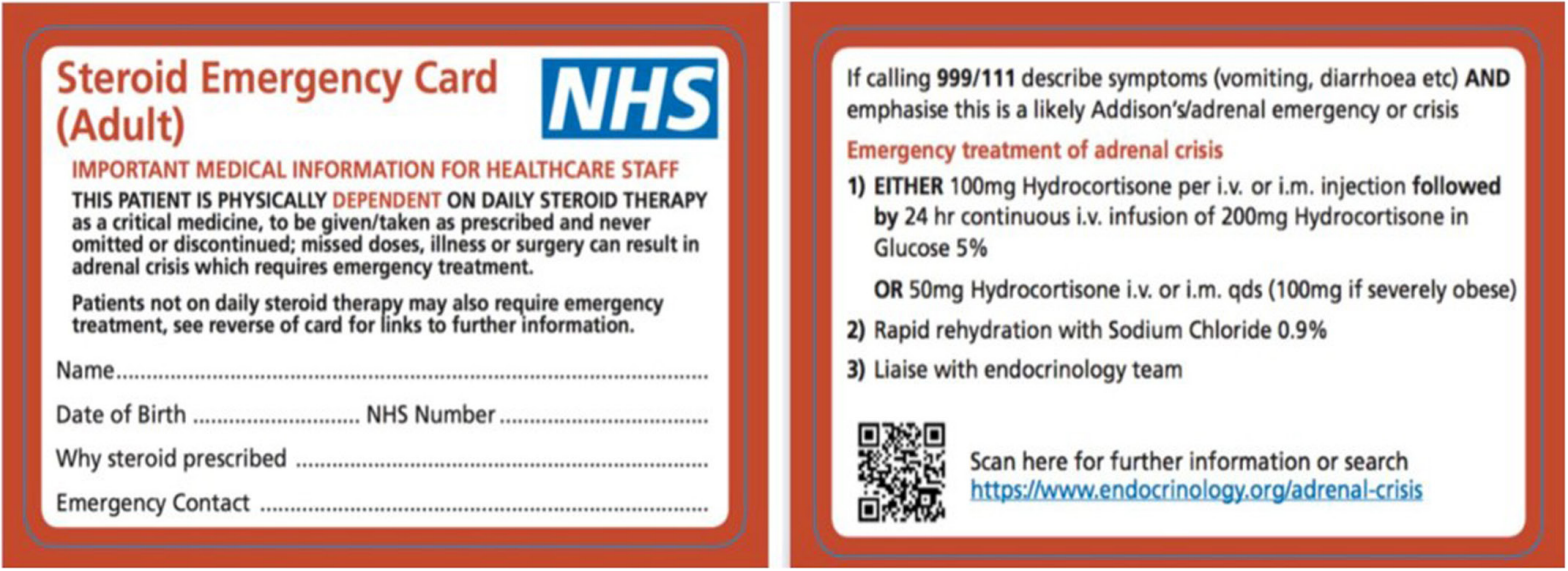
Steroid emergency card for patients with adrenal insufficiency issued by the UK National Health Service in March 2020 (downloadable at https://www.endocrinology.org/media/3563/new-nhs-emergency-steroid-card.pdf).

- Ensure that all patients are in possession of a ‘steroid emergency card’ or equivalent written instructions for healthcare staff on how to treat the patient in a major stress situation that prevents self-management. Figure 1 shows the recently issued UK version of the steroid card, developed further from a version originally proposed by a Swedish group (18) and further developed by the European Society of Endocrinology (https://adrenals.eu/emergency-card/). The UK version is downloadable at https://www.endocrinology.org/media/3563/new-nhs-emergency-steroid-card.pdf and includes a QR code that guides healthcare staff to a website with detailed instructions on how to manage a patient suffering from adrenal crisis (https://www.endocrinology.org/adrenal-crisis).

### B. Acute suspected or confirmed COVID-19 infection

**Figure 2:**
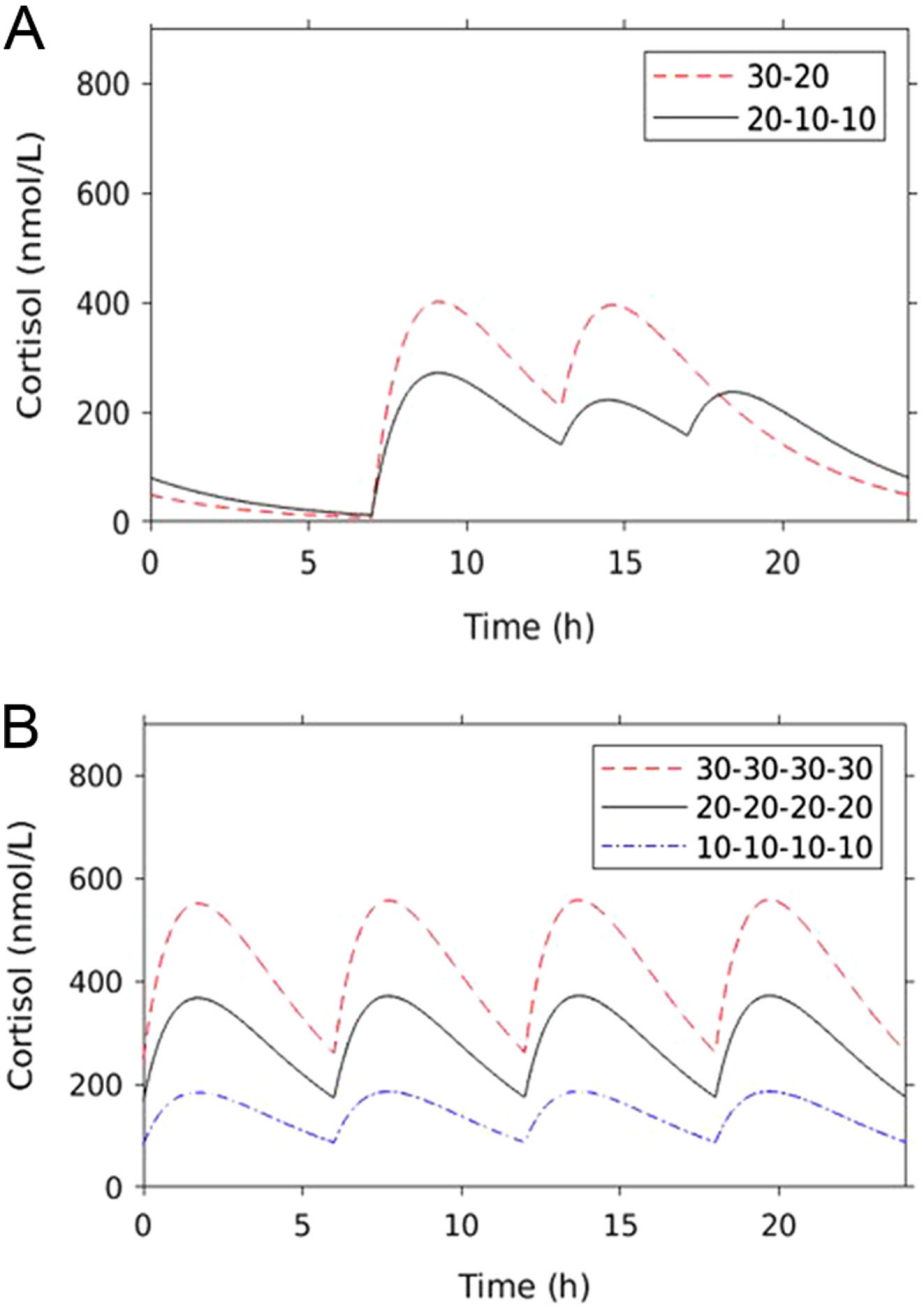
Prediction of 24-h oral hydrocortisone delivery dependent on timing and dosing. Prediction based on parameter estimates of circulating cortisol concentrations in a three-compartment model after (A) doubling of immediate release hydrocortisone doses administered at the usual times of two typical routine hydrocortisone regimens (two and three daily doses, respectively) and (B) oral administration of four equal doses in evenly spaced, 6-h intervals. This illustrates that the 6-h once administration ensures steady delivery of cortisol, while increasing hydrocortisone at the usual administration times results in a long stretch of time without appropriate hydrocortisone cover. For details on the employed modelling approach see (19); figure reproduced with permission.

**Figure 3:**
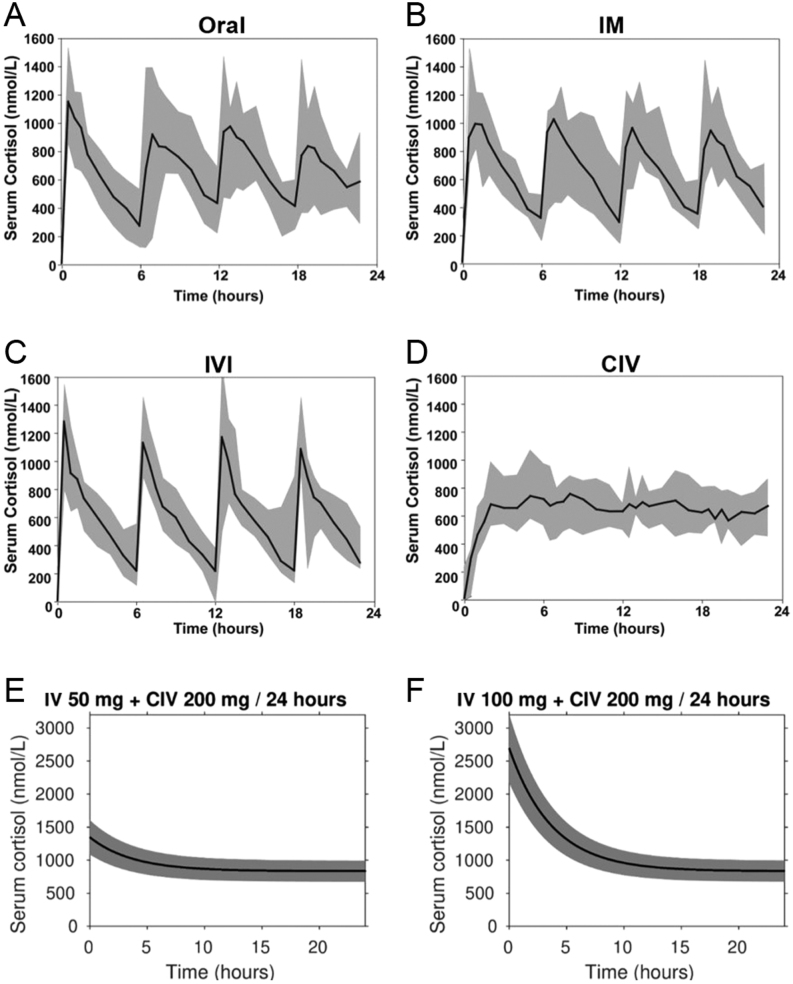
(Panels A, B, C and D) Serum total cortisol (nmol/L) in ten otherwise healthy, unstressed adult patients with adrenal insufficiency who paused their regular replacement and underwent frequent serum sampling after administration of 200 mg hydrocortisone/24 h in four different administration modes: 50 mg orally every 6 h (ORAL), 50 mg per i.m. bolus injection every 6 h (IM), 50 mg per i.v. bolus injection (IVI) and via continuous i.v. hydrocortisone infusion of 200 mg/24 h (CIV). Data are presented as median (black line) and range (shaded grey area). (Panels E and F) Linear pharmacokinetic modelling, based on serum cortisol measurements by tandem mass spectrometry after i.v. IVI and CIV hydrocortisone administration, to predict expected serum cortisol concentrations after 50 mg (E) and 100 mg (F) i.v. bolus injection, each followed by CIV infusion of 200 mg hydrocortisone/24 h. Figure modified after (20); reproduced with permission.

- ‘If a patient with adrenal insufficiency develops signs and symptoms suggestive of COVID-19’ (e.g. fever >38°C (or >100 F), a new or continuous dry cough, sore throat, loss of sense of smell or taste, aches and pains, and/or severe fatigue), they should seek like any other patient medical advice regarding the management of their suspected or confirmed COVID-19 infection, either over the internet (e.g. the UK online coronavirus service https://111.nhs.uk/covid-19/) or by a phone call to their general practitioner. As recommended for all affected by COVID-19, patients should rest and counteract the fever by taking 1000 mg doses of paracetamol every 6 h (with appropriate dose adjustment in children). They should try to keep well hydrated by drinking regularly, even during the night, ideally noting the amount of fluid they drink. They should monitor how much urine they pass; the excretion of only little amounts of dark, concentrated urine indicates insufficient hydration, which should prompt further increased oral fluid intake.
  Importantly, patients with adrenal insufficiency and an acute suspected or confirmed COVID-19 infection should also ‘immediately take a double hydrocortisone morning dose and then increase their hydrocortisone replacement to 20 mg four times daily’, that is, 20 mg hydrocortisone every 6 h, for example, at 0600 h, 1200 h, 1800 h and 2400 h (Table 1). In children, their usual daily dose should be trebled and administered orally in four equal doses every 6 h. Patients who normally take modified release hydrocortisone preparations should switch to taking 20 mg immediate release hydrocortisone every 6 h. Patients on prednisolone doses of 5–15 mg daily should immediately take 10 mg prednisolone every 12 h; patients on daily prednisolone doses >15 mg should continue to take their usual daily prednisolone dose but split it into a morning and late afternoon dose of at least 10 mg each time. Once the patient no longer has fever and starts to show significant clinical improvement, the 6-h once oral administration of 20 mg hydrocortisone can be tapered back to double dose of the routine replacement regimen and then normal routine doses once fully recovered. Asymptomatic patients who tested COVID-19 positive, for example, due to family screening, do not need to increase their routine replacement dose.
  While guidelines usually recommend doubling of the regular glucocorticoid replacement dose during intercurrent illness (15, 16, 17), the personal experience of the authors is that an acute COVID-19 infection is associated with significant and persistent acute inflammation and often continuous high fever, which in our view requires a more evenly spaced glucocorticoid cover throughout day and night. We have based our suggested doses on a three-compartment model of oral hydrocortisone delivery (19) (Fig. 2), drawing from experimental data from the Prevention of Adrenal Crisis in Stress (PACS) study (20) and a previous study on oral hydrocortisone pharmacokinetics (21). This modelling indicates that the mere doubling of the regular glucocorticoid dose could leave patients with prolonged periods of glucocorticoid deficiency during an acute and highly inflammatory infection such as COVID-19 (Fig. 2).
- Under no circumstances should patients hesitate to contact medical emergency services, ‘if the clinical signs and symptoms of COVID-19 significantly worsen’. Patients (or their carers) should ‘contact medical emergency services without delay and immediately administer their hydrocortisone emergency injection (100 mg i.m.)’. If for any reason they cannot administer the injection, they should immediately take 50–100 mg hydrocortisone orally, if possible, while waiting for medical emergency services to arrive. If need be, patients and their carers should consider making their own way to hospital and continue to take 50 mg hydrocortisone every 6 h. ‘Signs and symptoms indicating clinical deterioration in patients affected by COVID-19’, which typically occur 7–10 days after onset of the first COVID-associated symptoms, include:
  - feeling very dizzy on sitting or standing
  - feeling very thirsty despite drinking regularly
  - feeling very cold
  - shaking uncontrollably
  - becoming drowsy, confused or difficult to wake up
  - developing vomiting or severe diarrhoea
  - increasing shortness of breath with fast breathing (respiratory rate >24/min) or difficulty speaking in complete sentences.
- Following emergency injection of 100 mg hydrocortisone by self-injection or medical emergency personnel, the patients should be maintained on ‘major stress dose hydrocortisone, that is, 200 mg over 24 h, preferably in the hospital setting administered via continuous i.v. infusion’, which ensures that intermittent troughs in cortisol levels are avoided (Fig. 3) (20). Alternatively, 50 mg hydrocortisone could be administered via i.v. or i.m. bolus injection every 6 h. Patients should receive generous fluid resuscitation with i.v. isotonic saline. Both continuous administration of hydrocortisone in major stress dose and fluid resuscitation are crucially important for patients with adrenal insufficiency.
- ‘Children’ are much less frequently severely affected by COVID-19. However, in case of an acute COVID-19 infection with clinical deterioration, children with adrenal insufficiency should receive an immediate parenteral injection of 50–100 mg/m^2^ hydrocortisone (usually 25 mg for infants, 50 mg for school children) (16). This should be followed by parenteral administration of 50 mg/24 h for infants and 100 mg/24 h for school children, preferably by continuous i.v. infusion, alternatively, split in four equal parenterally administered doses every 6 h. Adolescents should be treated with adult doses.
- Patients with COVID-19 requiring mechanical ventilation: Acute respiratory distress syndrome (ARDS) is frequently observed in patients with COVID-19 infection requiring treatment in an intensive care unit (22, 23). It is a matter of debate (and subject of the ongoing RECOVERY trial in the United Kingdom) whether high dose glucocorticoid treatment is an effective therapy for COVID-19 related ARDS. However, patients with adrenal insufficiency receive glucocorticoids not as a pharmacological treatment, but as a life-saving replacement therapy in adequate doses to cover major stress. Therefore, if adrenal insufficiency patients develop COVID-19-related ARDS, their glucocorticoid replacement should never be interrupted, but continued at a major stress dose (200 mg/24 h) until mechanical ventilation is no longer required and significant clinical improvement has occurred. Then, gradual tapering of the glucocorticoid replacement dose in line with the observed further clinical improvement can be undertaken (Table 1).
- Co-incident type 1 diabetes is found in around 10% of patients with PAI (24, 25, 26). The clinical experience is that diabetic patients affected by COVID-19 quickly struggle to maintain glycaemic control, with significantly increased insulin requirements, and are more prone to diabetic ketoacidosis. A recent guidance on managing ‘type 1 and type 2 diabetes in COVID-19 patients’ (https://abcd.care/resource/concise-advice-inpatient-diabetes-during-covid19-front-door-guidance), advises stopping Dipeptidyl peptidase-4 (DPP4) inhibitors in patients with type 2 diabetes with an acute COVID-19 infection. DPP4 acts as a receptor for a subset set of corona viruses cell entry, similar to ACE2 (27); however, just like for ACE inhibitors, there is currently insufficient scientific evidence to judge whether use of DPP4 inhibitors impacts adversely on type 2 diabetes patients with COVID-19 (28).

### C. Regular monitoring of patients with adrenal insufficiency during the COVID-19 crisis

- We recommend that otherwise healthy patients with established adrenal insufficiency on routine steroid replacement should continue to be regularly monitored during prolonged COVID-19 lock-down periods, which may come with restricted access to healthcare facilities. These patients would normally undergo follow-up review in intervals of 6–12 months; during the COVID-19 crisis, these reviews could be undertaken by telephone or videoconferencing.
- Patients on stable replacement usually undergo annual checks of electrolytes and plasma renin to ensure adequacy of mineralocorticoid replacement, but during the COVID-19 crisis blood checks should be reserved for patients with clinical signs of hypotension, such as dizziness when standing up. Blood pressure self-measurement, for example, after sitting for at least 5 min and then again after standing up for a minute, should be encouraged; patients should also be taught how to take their heart rate and be advised which readings should prompt to contact their specialist care team for further advice (such as resting heart rate >100/min and systolic blood pressure <100 mmHg; otherwise healthy and well patients to remeasure after 1 h before contacting medical staff). Many healthcare centres have established bloodletting centres in convenient locations away from hospitals that can be used if a blood test is considered urgent.
- Routine glucocorticoid replacement therapy is monitored based on the patient's clinical performance and ability to cope with daily stress and does not require laboratory evaluation (29), thus history taking and discussions can easily take place via teleconferencing.

## Disclaimer

Due to the emerging nature of the COVID-19 crisis, this document is not based on extensive systematic review or meta-analysis, but on rapid expert consensus. The document should be considered as guidance only; it is not intended to determine an absolute standard of medical care. Healthcare staff need to consider individual circumstances when devising the management plan for a specific patient.

## Declaration of interest

Wiebke Arlt is the Editor-in-Chief of the European Journal of Endocrinology. W A was not involved in the review or editorial process for this paper, on which she is listed as an author. The other authors have nothing disclose.

## Funding

This guidance did not receive any specific grant from any funding agency in the public, commercial or not-for-profit sector.
